# TBL1X and Flot2 form a positive feedback loop to promote metastasis in nasopharyngeal carcinoma

**DOI:** 10.7150/ijbs.68091

**Published:** 2022-01-01

**Authors:** Hongjuan Xu, Xuejun Yan, Hecheng Zhu, Yuanbo Kang, Weiren Luo, Jin Zhao, Kefan Zhou, Xiwu Liu, Li Ye, Quanwei Zhou, Shasha Li, Ming Zhao, Lei Wang, Bin Zhu, Weidong Liu, Jianxiong Li, Xingjun Jiang, Caiping Ren

**Affiliations:** 1 Cancer Research Institute, Department of Neurosurgery, National Clinical Research Center for Geriatric Disorders, NHC Key Laboratory of Biological Nanotechnology, Xiangya Hospital, Central South University, Changsha, Hunan 410008, China.; 2 NHC Key Laboratory of Carcinogenesis and the Key Laboratory of Carcinogenesis and Cancer Invasion of the Chinese Ministry of Education, School of Basic Medical Science, Central South University, Changsha, Hunan 410008, China.; 3 Changsha Kexin Cancer Hospital, Changsha, Hunan 410008, China.; 4 Cancer Research Institute, Shenzhen Third People's Hospital, the Second Affiliated Hospital of Southern University of Science and Technology, Shenzhen, Guangdong 518112, China.; 5 Department of Neurosurgery, National Clinical Research Center for Geriatric Disorders, Xiangya Hospital, Central South University, Changsha, Hunan 410008, China.; 6 Department of Radiotherapy, Chinese PLA General Hospital, Beijing 100089, China.

**Keywords:** Nasopharyngeal carcinoma (NPC), metastasis, prognosis, Transducin β-like protein 1 X (TBL1X), Flotillin-2 (Flot2)

## Abstract

Metastasis is the main cause of death in patients with nasopharyngeal carcinoma (NPC). The molecular mechanisms underlying the metastasis of NPC remain to be elucidated. TBL1X has been shown abnormally expressed in diverse cancers. However, the role and mechanism of TBL1X in NPC remain unknown. Here, we showed TBL1X expression was significantly higher in metastatic NPC tissues compared to non-metastatic tissues and significantly correlated with TNM stage and metastasis of NPC patients. In addition, NPC patients with high TBL1X expression had a poor prognosis. TBL1X interacted with TCF4 to trans-activate Flot2 expression. TBL1X promoted NPC cell migration and invasion in vitro and *in vivo* through Flot2. Moreover, Flot2 increased the expression of TBL1X by upregulating c-myc, which was identified to be a positively regulatory transcription factor of TBL1X. TBL1X could restore the functional changes of NPC cells resulting from Flot2 alteration. TBL1X and Flot2 were positively correlated in NPC. Patients with high expression of both TBL1X and Flot2 possessed poorer overall survival (OS) and disease-free survival (DFS) compared to patients with high expression of any single one of the two proteins. Our findings demonstrate that TBL1X and Flot2 positively regulate each other to promote NPC metastasis, which provides novel potential molecular targets for NPC treatment.

## Introduction

Nasopharyngeal carcinoma (NPC) is a common head and neck cancer originating from the nasopharyngeal epithelium with about 133,354 new cases and 80,008 related death cases in 2020^1^. Compared with other cancers, NPC shows the uneven geographical distribution, with approximately 70% of new cases occurring in east and southeast Asia^2^. Because of the concealed anatomical location and lack of specific symptoms during early stages, 70% of NPC patients are typically diagnosed with locoregionally advanced disease at the initial diagnosis^3^. Metastasis is the primary cause of death in NPC patients^4^, thus, it is critical to elucidate the molecular mechanisms of NPC metastasis to identify more effective treatment avenues.

TBL1X is encoded by the *TBL1X* gene located in Xp22.3 genomic region, which contains F-box-like and WD-40-repeat domains, and is identified as a core component of the co-repressor silencing mediator of retinoid and thyroid hormone receptor (SMRT)-nuclear receptor co-repressor (N-COR) complex^5^, ^6^. It has been reported that TBL1X acts as an oncogene to promote the progression of multiple tumors by directly regulating different proteins' expression. For example, TBL1X promotes EMT of breast cancer cells by acting as a cofactor of ZEB1 to regulate the expression of CDH1, and the high expression of TBL1X contributes to metastasis of breast cancer and is correlated with the poor prognosis of patients with breast cancer^7^. Tegavivint suppresses chemotherapy-resistant and metastatic osteosarcoma by disturbing β-catenin-TBL1 binding^8^, and SUMOylation of TBL1/TBLR1 promotes the migration and invasion of bladder cancer cells^9^. In pancreatic ductal adenocarcinoma, TBL1X acts as a malignancy checkpoint 10. Nevertheless, the role of TBL1X in NPC metastasis remains unclear.

Flotillin-2 (Flot2) is a lipid raft protein and has been shown to regulate the cancer cell cycle, proliferation, migration, and invasion, which are key processes in cancer development^11^, ^12^. Upregulation of Flot2 occurs in glioma^13^, ^14^, breast cancer^15^, NPC^16^, melanoma^17^, hepatocellular carcinoma^18^, lung cancer^19^, and oral squamous cell carcinoma^20^. In the previous study, we used suppressive subtractive hybridization (SSH) to identify that Flot2 is a differentially expressed gene between 5-8F (highly tumorigenic and metastatic) and 6-10B (tumorigenic and non-metastatic) NPC cells, with markedly higher expression in 5-8F cells. Moreover, we found that Flot2 promotes NPC development by activating the NF-κB and PI3K/Akt3 signaling pathways, and its up-regulated expression induces abnormal cancer cell cycle, apoptosis, migration, and invasion characteristics^21^. However, further studies are needed to elucidate the mechanisms underlying the functions and regulation of Flot2 in NPC.

In this study, we demonstrated the pro-metastatic effects of TBL1X and Flot2 in NPC both in vitro and *in vivo* and identified the mutual regulation relationship between TBL1X and Flot2. High expression of TBL1X and Flot2 was correlated with reduced survival of NPC patients. Our results suggest that the TBL1X-Flot2 feedback axis contributes to NPC progression and might be a potential molecular target for NPC treatment.

## Materials and Methods

### Data acquisition and processing

To detect the mRNA level of TBL1X in 33 types of cancers, we downloaded the mRNA sequencing data using UCSC Xena (https://xena.ucsc.edu/), containing 33 tumors and 30 types of normal tissues. The expression of TBL1X was evaluated in 30 normal tissues and 33 tumors. Data analysis was conducted using R software (Version 4.0.3) and using “ggpubr” to draw pictures. The expression array data (GSE53819) containing 18 NPC tissues and 18 control tissues, GSE12452 containing 31 NPC tissues and 10 control tissues, and GSE64634 containing 12 NPC tissues and 4 control tissues were downloaded from the Gene Expression Omnibus Database (GEO) (https://www.ncbi.nlm.nih.gov/geo/) for subsequent analysis. After gene reannotation and batch normalization, a new GEO batch containing the three GEO datasets (61 NPC samples, 32 control tissues) was formed and analyzed together.

### Tissue specimens

Firstly, the fresh clinical samples of NPC (n=40) were collected from Xiangya Hospital, Central South University. All patients wrote the informed consent. To further investigate the expression of TBL1X and Flot2 in NPC, tissue microarrays of NPC (n=126) were obtained from Shanghai Super Biotek (Shanghai, China) with permission of the local Institutional Review Board.

### Immunohistochemistry (IHC)

The paraffin-embedded NPC tissues were sectioned into 5 µm-thick slides. The slides were deparaffinized, performed for antigen retrieval, and blocked with a proper blocking solution. The slides were then incubated with the primary antibody against TBL1X (1:50, #sc137006, Santacruz) or Flot2 (1:50, #28208-1-AP, Proteintech). After that, the slides were incubated with relative secondary antibodies and stained with a DAB staining kit (#ZLI9017, ZSGB BIO, Beijing, China).

### Cell lines and cell culture

Human NPC cell lines (5-8F, 6-10B, c666-1, HK-1, SUNE1, HONE1) were cultured in RPMI 1640 medium supplemented with 10% fetal bovine serum (FBS) and antibiotics (100 U/ml penicillin and 0.1mg/ml streptomycin). Normal nasopharyngeal epithelial cells NP69 were cultured in KSFM medium, and normal nasopharyngeal epithelial cells NP460 were cultured in KSFM and Cascade Biologics EpiLife medium (50%:50%). All cell lines were incubated at 37°C with 5% CO_2_ in a humidified atmosphere.

### Gene silencing and overexpression

Endogenous Flot2 and TBL1X were silenced by small hairpin RNA constructs (pLV-shFlot2, pLV-shTBL1X), and the target sequences are as follows: Flot2: TGTTGTGGTTCCGACTATAAA (1#), GGTTGTGCAGCGCAAGAAA (2#); TBL1X: ATGATCTTCAGGCTCACAATA (1#), CTTCGACAAGTGCGTCCATAT (2#); TCF4: CACGAAATCTTCGGAGGACAA (1#), CAACGGGACAGACAG TATAAT (2#). pLV-oeFlot2 and pLV-oeTBL1X lentiviral expression plasmids were constructed. pLV-control, pLV-shFlot2, pLV-shTBL1X, pLV-oeTBL1X, and pLV-oeFlot2 plasmids were transfected into 293T cells with psPAX2 and pMD2G plasmids by using Lipofectamine 2000 (Invitrogen). Then, the supernatant of the medium was collected and filtered through a 0.45 μm filter. Subsequently, we used the supernatant to infect the cancer cells with polybrene. After 48 h, the positive and stable cell lines were obtained by screening with 2 μg/ml of puromycin.

### Western blotting analysis

Total protein lysates were incubated in RIPA strong lysis buffer with 1% protease inhibitor cocktail and phosphatase inhibitor for 30 min on ice, then the supernatant was collected by centrifuging for 30 min at 4℃, 12,000 rpm. The concentration of proteins was measured with the BCA method. 40 μg total proteins were separated by using 10%-12% sodium dodecyl sulfate-polyacrylamide gel electrophoresis (SDS-PAGE). The protein bands were transferred from gel to polyvinylidene difluoride (PVDF) membranes, and the membranes were blocked for 2 h with 5% BSA in Tris buffer saline with 0.1% Tween 20 (TBST) buffer at room temperature. Then, the membranes were incubated with primary antibodies at 4°C overnight and washed with TBST buffer (3 times, 6 min each) on the next day. The membranes were subsequently incubated with relevant secondary antibodies for 2 h at room temperature and visualized with a chemiluminescence reagent (Biosharp). The following antibodies from various vendors were used for studies: anti-TBL1X (1:500, #13540-1-AP, Proteintech); anti-GAPDH (1:1000, #SC-47724, Santa Cruz); anti-Flot2 (1:1000, #3426, Cell Signaling Technology); anti-E-cadherin (1:5000, #20874-1-AP, Proteintech); anti-c-myc (1:500, #10828-1-AP, Proteintech); anti-TCF4 (1:1000, #2569, Cell Signaling Technology); anti-rabbit IgG (A0545) and anti-mouse IgG (A9044) (1:40,000, Sigma).

### Quantitative real-time polymerase chain reaction (qRT-PCR)

Total RNA was extracted from cells with the TRIZOL method. 1 μg of RNA were used to synthesize cDNA using RevertAid First Strand cDNA Synthesis Kit (#K1622, Thermo Scientific). SYBR Green Mix (#Q111-02, Vazyme) was used for qRT-PCR detection, and GAPDH was used as an internal control. All samples were analyzed in triplicate and the data were calculated according to the 2 ^-ΔΔCT^ method. The used primers are as follows:

GAPDH forward primer: 5'-CCAGCAAGAGCACAAGAGGAA-3';

GAPDH reverse primer: 5'-ATGGTACATGACAAGGTGCGG-3';

TBL1X forward primer: 5'-CAGGGCTCCTTATGGTGACT-3';

TBL1X reverse primer: 5'-CATATCAGATGCCTCGCAGA-3';

Flot2 forward primer: 5'-GGCTTGTGAGCAGTTTCTGG-3';

Flot2 reverse primer: 5'-TCGAAGGCTCGCTTAGAGTC-3'.

### Transwell matrigel invasion assay

24-well transwell plates (8-μm pore size, Corning) were used for cell invasion assay. 20,000 NPC cells suspended in serum-free medium were plated in the upper chambers coated with matrigel (BD Biosciences), and 0.6 ml of RPMI 1640 medium with 10% FBS was added to the lower chamber. After incubation for a suitable amount of time, the invaded cells were fixed in 4% paraformaldehyde, stained by crystal violet, and counted under a microscope.

### Wound-healing migration

For wound-healing migration assays, the cell monolayers were mechanically disrupted using a sterile 200 μl pipette tip to generate a linear wound. The average area between the two margins of the wound was measured using Image J Software at suitable time points.

### Chromatin immunoprecipitation (ChIP) assay

ChIP assays were conducted using the ChIP Kit (Abcam 500) according to the manufacturer's protocol. We used the following antibodies to immunoprecipitate the chromatin: anti-TBL1X (#SC137006, Santacruz); anti-TCF4 (#2569, Cell Signaling Technology); anti-c-myc (#10828-1-AP, Proteintech). The precipitated DNA fragments were used to detect the protein-DNA binding results by qPCR as mentioned above and agarose gel electrophoresis (150V, 45min). The relative ChIP primers are as follows:

Flot2 forward primer: 5'-GGGCACCAAGAGGGGGTGCCCAGAC-3';

Flot2 reverse primer: 5'-ATCGGGTTCAGCATCCTCCCCAGGC-3';

TBL1X forward primer 1: 5'-GTTGCAAGCAGATTCTTG-3';

TBL1X reverse primer 1: 5'-CAAGGAGGTAGTGTATTCTGAGA-3';

TBL1X forward primer 2: 5'-AGGAGAATCAATCAACCACAATG-3';

TBL1X reverse primer 2: 5'-CAAGGAGTTCCAGAGCGAGAG-3'.

### Co-immunoprecipitation (Co-IP) assay

Total proteins were extracted by NP-40 buffer as mentioned above. The concentration of proteins was measured, and the samples were respectively divided into three parts (Input, IgG, IP). Each part contained about 1-2 mg protein. 20 μl protein A/G beads (#B23201, Bimake, USA) were added to each of the sample parts and the mixture was incubated for 2 h at 4℃. Then, IgG or IP antibodies were added to the samples, and the mixture was incubated overnight at 4℃. The next day, 20 μl protein A/G beads were added to the samples again and incubated together for 2 h at 4℃. Magnetic beads were collected, mixed with NP40 lysis and loading buffer, and denatured at 95℃ for 5 min. Then the supernatant was collected by centrifugation. At last, protein-protein interaction was detected by western blotting as mentioned above.

### Immunofluorescence staining

Cells were added to the coverslip in 24 well plates. The next day, cells were fixed with 4% paraformaldehyde for 5 min. 5% FBS was used to incubate cells for 2 h at RT. The cells were incubated with primary antibodies overnight at 4℃, and then incubated with relative FITC or Cy5 labeled secondary antibodies at room temperature for 2 h in dark. DAPI solution was used to stain the cell nuclear. The staining photos were taken by using Laser Scanning Confocal Microscopy (UltraView, Perkin Elmer, Cambridge, UK).

### Dual-luciferase assay

The TBL1X and Flot2 promoter regions including -2000 bp upstream of the transcription start site or relevant mutant sequences were cloned into the pGL3-basic vector. A dual-luciferase system (Promega, USA) was used to measure firefly and renilla luciferase activities according to the manufacturer's protocol.

### Mouse lung metastasis model

All of the animal experiments were approved by the Animal Care Committee of Central South University following Institutional Animal Care and Use Committee guidelines. To study the effects of Flot2 and TBL1X on NPC metastasis *in vivo*, the 18-20 g male nude mice (BALB/C) used in this study were purchased from the Animal Research Center of Central South University. To establish the *in vivo* lung metastasis model, 200 μL blank RPMI 1640 containing 2×10^6^ 5-8F control, 5-8F shTBL1X, 5-8FshTBL1X+oeFlot2, 5-8FshFlot2, or 5-8FshFlot2+oeTBL1X cells were respectively injected into the mice by the tail vein injection (n=6 per group). After about 2 months, the mice were sacrificed and their lung tissues were detached. All of the dissected tissue samples were paraffin-embedded, sectioned, and stained with HE, and the lung metastases were counted.

### Statistical analysis

All the experimental data were presented as (means ± SD). Analyses were performed by using GraphPad Prism Software 8.0 (San Diego, CA, USA) and SPSS 19.0. Student *t*-test was used for comparing two groups, and One-way ANOVA was applied for comparing multiple groups. Pearson's χ^2^ test was used to analyze the relevance of TBL1X and Flot2 expression with clinicopathological characteristics. Spearman correlation coefficient was used to assay the correlation between TBL1X and Flot2. Kaplan-Meier analysis was performed to produce OS and DFS curves, and the log-rank test was used to calculate *P*-values. ^*^P <0.05, ^**^P <0.01 and ^***^P <0.001 were considered statistically significant.

## Results

### TBL1X expression and clinical significance in NPC

**Figure 1:**
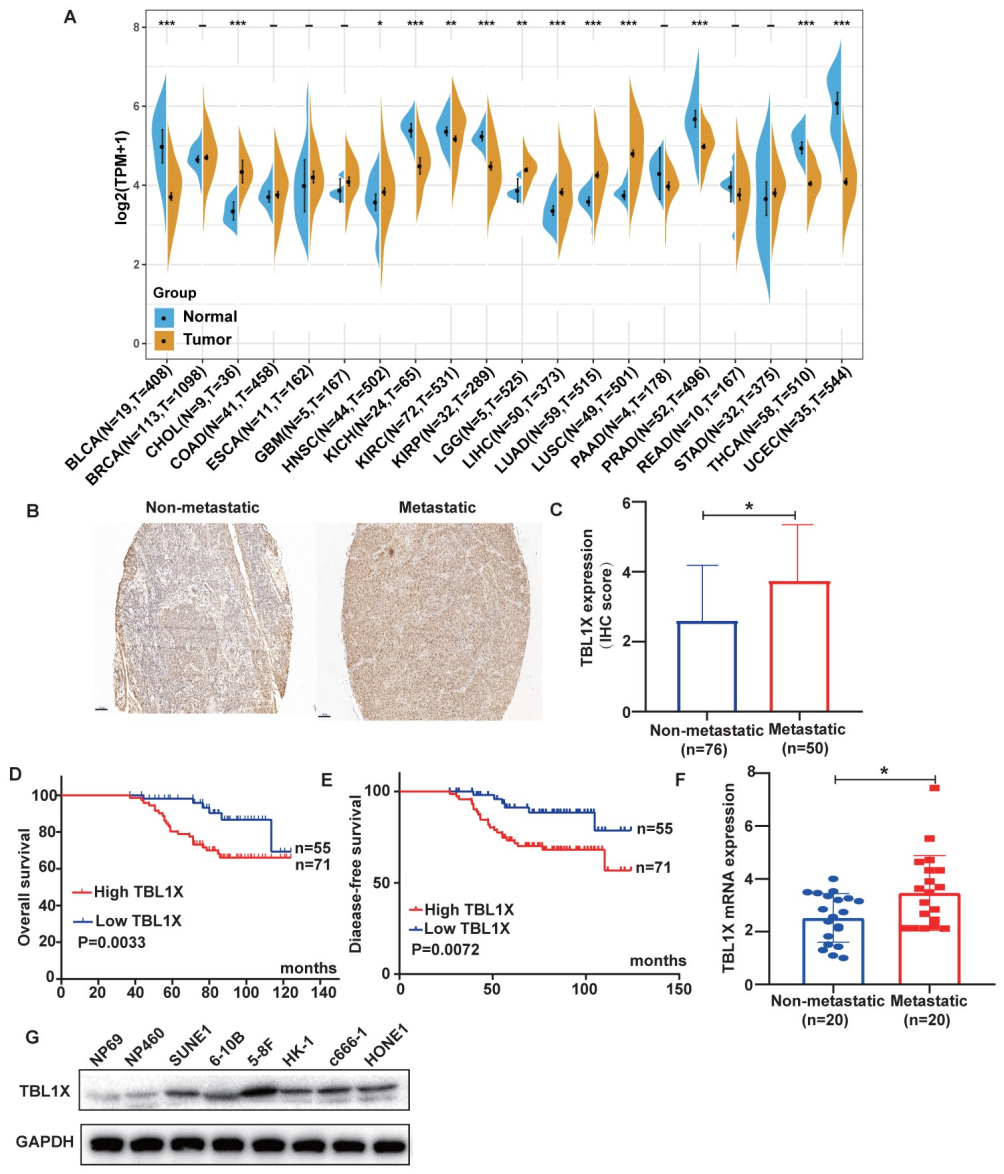
** The expression of TBL1X in NPC and its correlation with patient survival**. (A) TBL1X expression in different cancers. (B) Representative images of IHC staining of TBL1X in non-metastatic and metastatic NPC tissues (scale bar=100 μm). (C) The IHC scores of TBL1X expression. (D, E) The OS (D) and DFS (E) curves of NPC patients were associated with TBL1X expression. (F) Analysis of TBL1X mRNA expression in NPC tissues. (G) TBL1X protein expression in NPC cells and normal NPE cells. The results are shown as means±SD. *P <0.05, **P <0.01, ***P<0.001.

Firstly, we carried out a pan-cancer analysis to investigate the expression of TBL1X in different cancer types and their corresponding normal tissues using TCGA datasets. The results demonstrated that TBL1X expression was significantly different in 13 types of tumors compared to their relative normal tissues, with higher expression of TBL1X in six types of cancers, including HNSC, and lower expression in the other seven types of tumor tissues (Figure 1A). NPC is a common form of HNSC, so we analyzed the expression of TBL1X in NPC microarray by IHC methods. The results showed TBL1X expression was positively correlated with the clinical stages (*P*<0.001) and metastasis of NPC (*P*<0.001) (Table 1). Meanwhile, NPC patients with metastasis possessed higher staining intensity than patients with non-metastasis (Figure 1B), and the scores of IHC in the metastatic NPC group were also significantly higher than the non-metastatic group (*P*<0.05) (Figure 1C). Patients with high TBL1X levels had reduced OS and DFS, compared to those with the low level of TBL1X (Figure 1D-E). Consistent with the IHC results, we detected the mRNA level of TBL1X in fresh NPC samples by qRT-PCR, and the results displayed that the expression of TBL1X was significantly higher in metastatic tissues than in non-metastatic tissues from NPC patients (*P*=0.0171) (Figure 1F). Western blotting showed that TBL1X was upregulated in NPC cell lines (5-8F, c666-1, 6-10B, HK-1, SUNE1, and HONE1), as compared to normal nasopharyngeal epithelial (NPE) cell lines (NP69 and NP460) (Figure 1G).

### TBL1X enhances migration and invasion of NPC cells by regulating the EMT pathway

**Figure 2:**
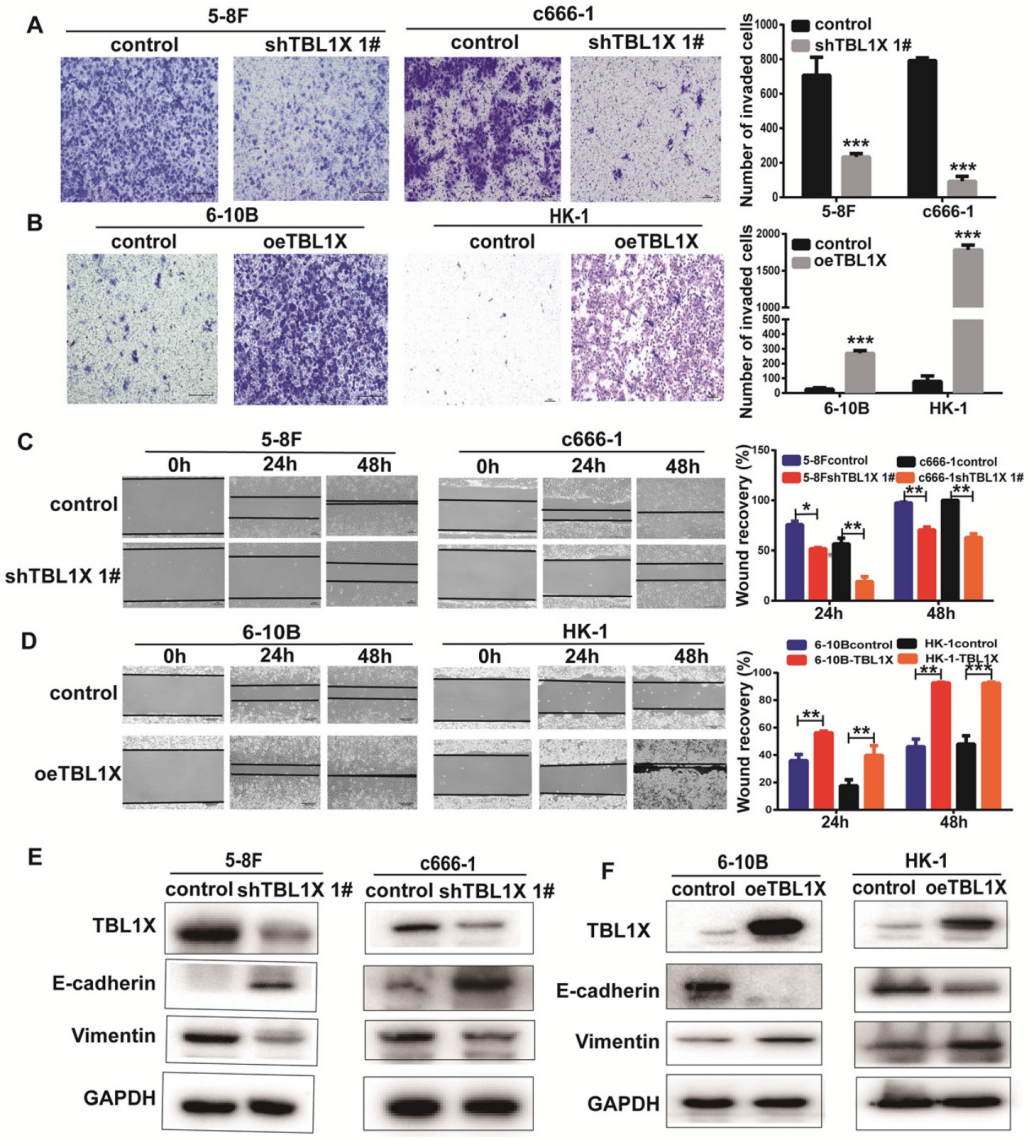
** TBL1X enhances NPC cell migration and invasion**. (A, B) Transwell invasion assay indicated the invasion abilities after knockdown (A) and overexpression (B) of TBL1X in NPC cells. (C, D) Wound-healing assay indicated the migration abilities after knocking down (C) and overexpression (D) of TBL1X in NPC cells. (E, F) Western blotting results indicated the effects of TBL1X interference (E) and overexpression (F) on EMT markers. The results are shown as means±SD. *P<0.05, **P<0.01, ***P<0.001.

To investigate the potential function of TBL1X in NPC cells, we established 5-8FshTBL1X, c666-1shTBL1X, 6-10B-TBL1X, and HK-1-TBL1X cell lines. Transwell invasion experiments showed that reduced TBL1X expression suppressed the invasion of 5-8F and c666-1 cells using two different siTBL1X sequences (Figure 2A, Supplementary Figure 1A), whereas TBL1X overexpression increased the invasion of 6-10B and HK-1 cells (Figure 2B). Scratch tests revealed that knockdown of TBL1X significantly reduced the migration of 5-8F and c666-1 cells (Figure 2C, Supplementary Figure 1B). Meanwhile, TBL1X overexpression enhanced the migration of 6-10B and HK-1 cells (Figure 2D). Consistent with these results, western blotting showed that TBL1X knockdown increased E-cadherin expression and decreased Vimentin expression (Figure 2E, Supplementary Figure 1C). By contrast, TBL1X overexpression resulted in decreased E-cadherin expression and increased Vimentin expression (Figure 2F). These results indicated that TBL1X affects the invasion and migration of NPC cells via altering the EMT pathway of NPC cells.

### TBL1X promotes EMT by enhancing Flot2 expression

**Figure 3:**
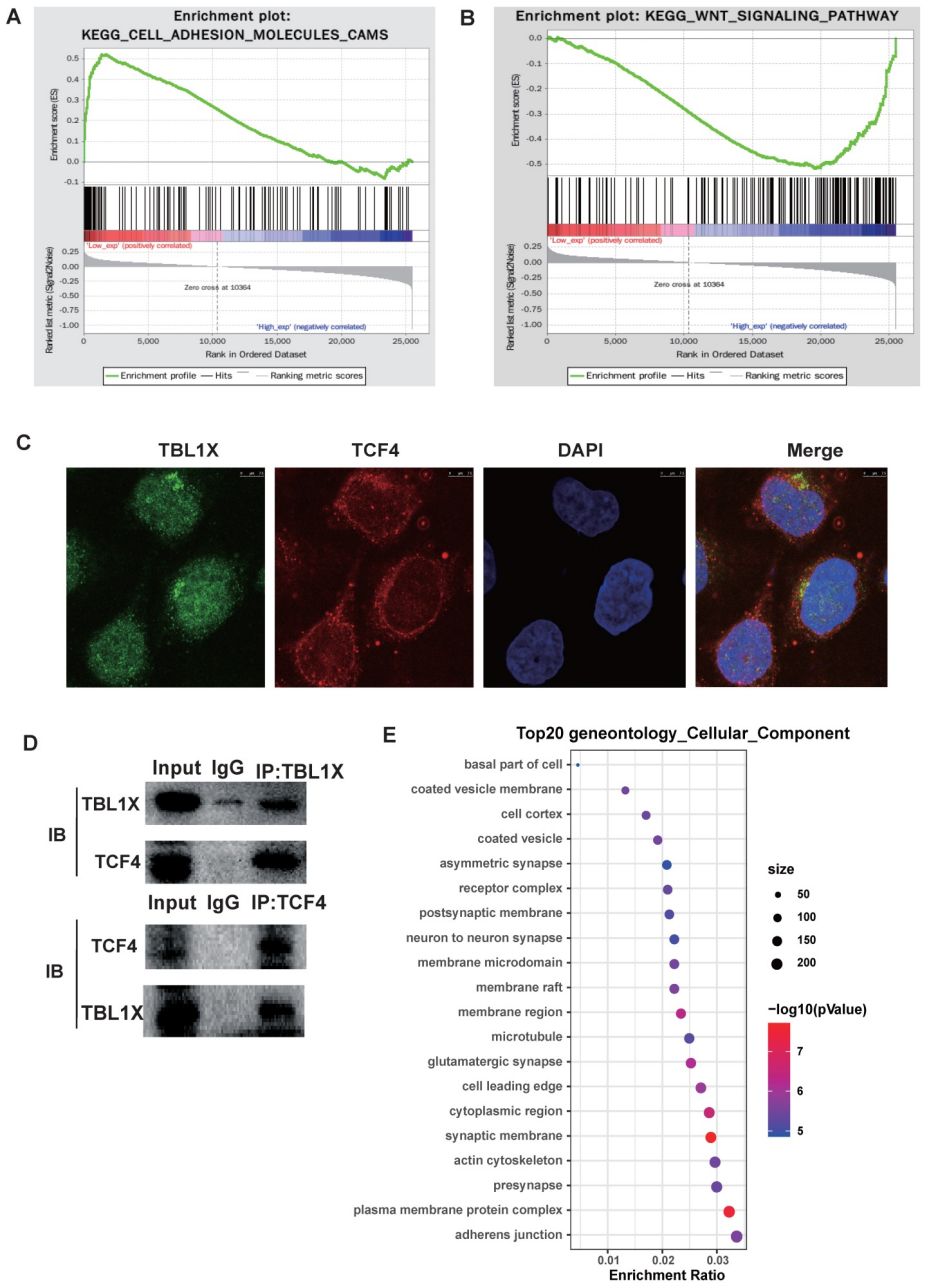
**Functional mechanisms of TBL1X in NPC**. (A, B) GSEA analysis of TBL1X expression. (C) Immunofluorescence staining showed that TBL1X and TCF4 colocalized in nuclear. (D) Co-IP test demonstrated that TBL1X and TCF4 bound with each other. (E**)** The top 20 enriched gene ontology cellular component based on KEGG enrichment analysis of TBL1X and TCF4 regulated genes.

To investigate the potential molecular mechanisms underlying the functions of TBL1X in NPC, we classified all tumor samples into high and low expression groups based on the median of TBL1X expression in the TCGA datasets. Firstly, we conducted GSEA to identify pathways that were differentially activated between low and high TBL1X expression groups. Results showed that low TBL1X expression significantly correlated with adhesion molecules (Figure 3A). Meanwhile, high TBL1X expression was positively related to the Wnt signaling pathway (Figure 3B). As a transcription factor, TCF4 is a key component of Wnt signaling. In this study, immunofluorescence staining and Co-IP experiments showed that TBL1X and TCF4 formed protein-protein complexes in NPC cells (Figure 3C-D). Therefore, TBL1X might regulate the expression of downstream genes along with TCF4. We screened out the possible genes that were regulated by TBL1X and TCF4 through CHEV, ENCODE, JASPAR, MotifMap, TRANSFAC websites. Then, we conducted a deeply functional enrichment analysis to explore the potential function of the related genes and found that among the top 20 terms of gene ontology cellular component, the most significant item was adhesion junction. Of interest, Flot2 was located in several GO terms, such as adhesion junction, plasma membrane protein complex, cytoplasmic region, membrane region, membrane raft, and membrane microdomain (Figure 3E). These results suggested that Flot2 might be involved in the TBL1X regulatory network.

**Figure 4:**
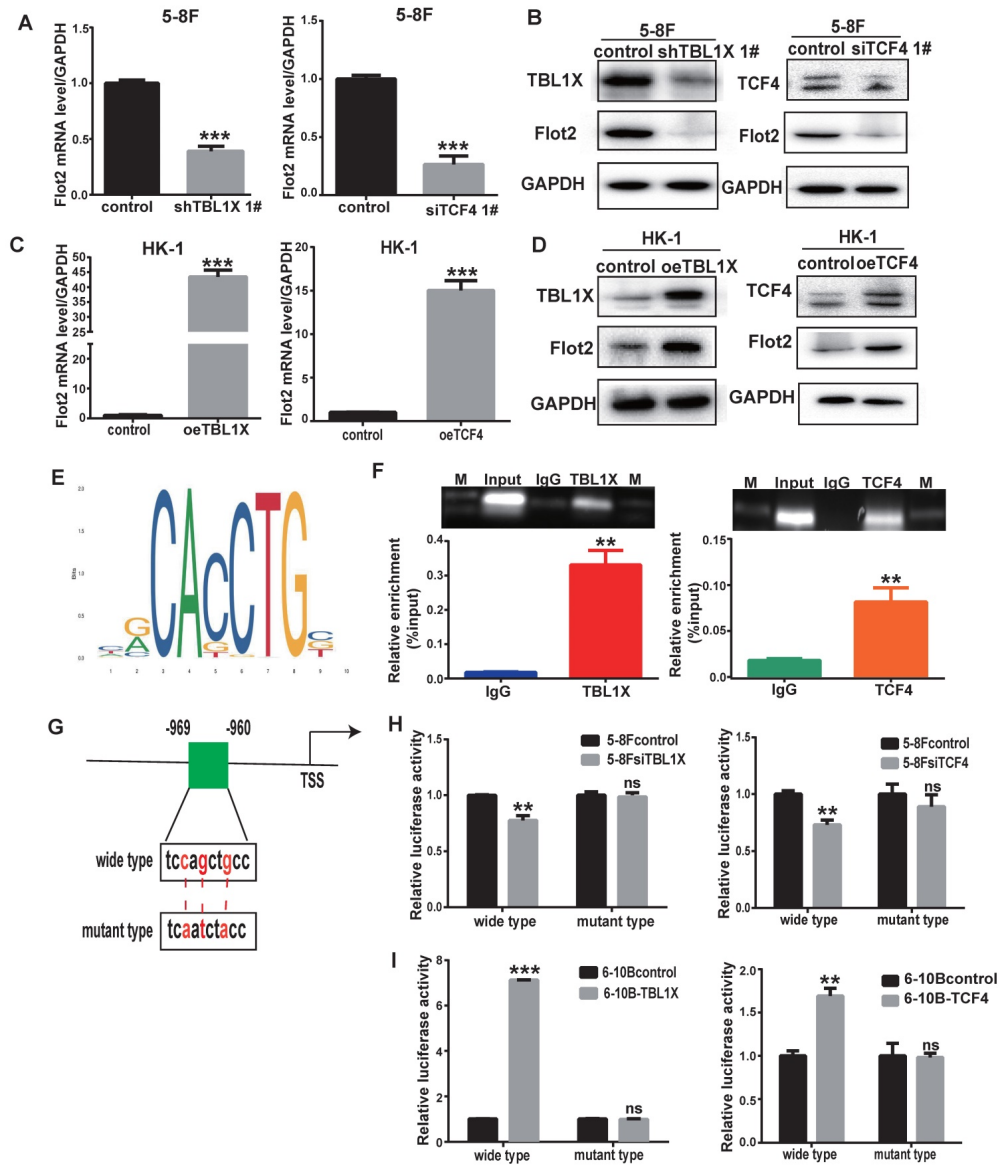
**TBL1X regulates Flot2 expression by binding to its promoter with TCF4**. (A, B) qRT-PCR (A) and Western blotting (B) showed that knockdown of TBL1X or TCF4 reduced the expression of Flot2. (C, D) qRT-PCR (C) and Western blotting (D) assays showed that overexpression of TBL1X or TCF4 enhanced the expression of Flot2. (E) JASPAR database predicted the binding sites of TCF4 in Flot2 promoter region. (F) ChIP experiments indicated that TBL1X and TCF4 could bind to the promoter region of Flot2. (G) Construction design of dual-luciferase reporter plasmids. (H) Relative luciferase activity in 6-10B cells transfected with pGL3basic-Flot2 plasmids containing wild type or mutant Flot2 promoter and treated with siTBL1X or siTCF4. (I) Relative luciferase activity in 5-8F cells overexpressing TBL1X or TCF4 after transfection of pGL3basic-Flot2 plasmids containing wild type or mutant Flot2 promoter. The results are shown as means±SD. *P<0.05, **P<0.01, ***P<0.001.

To further determine the role of TBL1X in Flot2 expression, we performed qRT-PCR and western blotting tests and the results demonstrated that both mRNA and protein levels of Flot2 were decreased after knockdown of TBL1X or TCF4 using two different siRNA sequences in 5-8F cells (Figure 4A-B, Supplementary Figure 2A-B). Overexpression of TBL1X or TCF4 led to increased Flot2 expression (Figure 4C-D). We then identified a consensus TCF4 binding site in the promoter region of Flot2 using the ALGGEN PROMO and JASPAR online tools (Figure 4E). ChIP assays in 5-8F cells were conducted using anti-TBL1X, anti-TCF4 antibodies, and IgG was used as a negative control antibody. The sequence containing the putative TCF4 binding site was amplified. As shown in Figure 4F, the TBL1X/TCF4 complex is directly bound to the Flot2 promoter region. The Flot2 promoter region fragments, including wild-type or mutated TCF4 binding sites, were inserted into pGL3-basic plasmids to construct gene reporter vectors (Figure 4G). Knockdown of TCF4 or TBL1X genes in 5-8F cells significantly decreased luciferase activity in cells transfected with wild-type Flot2 promoter plasmids, but not in cells with mutant Flot2 plasmids (Figure 4H). After transfecting TBL1X or TCF4 expressing plasmids, the luciferase activity was significantly increased in 6-10B cells transfected with wild-type Flot2 promoter plasmids, but not in cells transfected with mutant Flot2 plasmids (Figure 4I). These results demonstrated that TBL1X could form a protein-protein complex with TCF4 to increase Flot2 expression at the transcriptional level by binding to the promoter region of the Flot2 gene.

**Figure 5:**
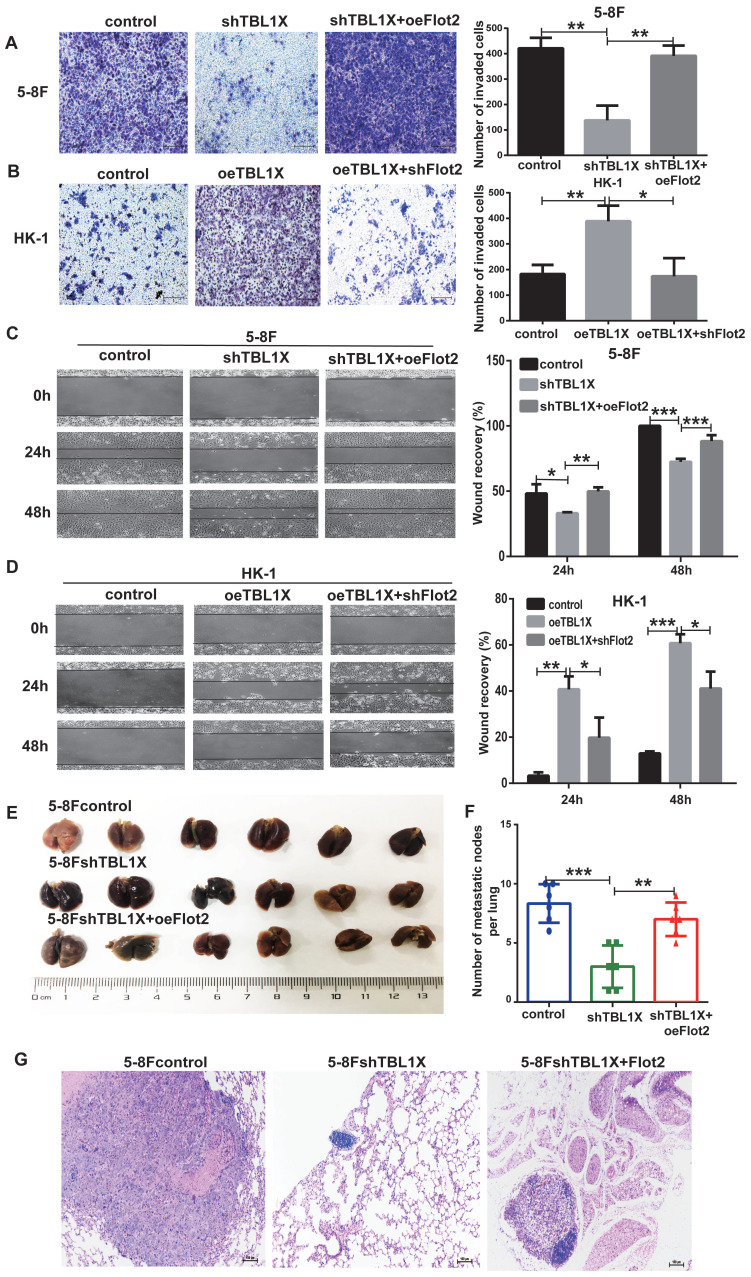
** Flot2 rescues the effects of altered TBL1X expression on NPC cells *in vitro* and *in vivo***. (A) Matrigel invasion analysis showed that oeFlot2 enhanced the invasion of 5-8FshTBL1X cells. (B) shFlot2 inhibited the invasion of HK-1-TBL1X cells. (C) Wound-healing assay showed that oeFlot2 enhanced the migration of 5-8FshTBL1X cells. (D) shFlot2 reduced the migratory ability of HK-1-TBL1X cells. (E) Nude mouse metastasis assay showed the lung metastases via tail vein injection of 5-8Fcontrol, 5-8FshTBL1X or 5-8FshTBL1X+oeFlot2 cells (n=6, respectively). (F) The number of lung metastases in mice. (G) Representative HE staining images of lung metastases resulting from 5-8Fcontrol, 5-8FshTBL1X and 5-8FshTBL1X+oeFlot2 cell inoculation. The results are shown as means±SD. *P<0.05, **P<0.01, ***P<0.001.

From the above experiments, it was shown that Flot2 was regulated by TBL1X. Thus, we analyzed whether Flot2 would reverse the effects of altered TBL1X expression on NPC cell functions. TBL1X knockdown cells were transfected with Flot2 expression plasmids. The results showed that Flot2 expression significantly increased the invasion and migration abilities of TBL1X knockdown cells (Figure 5A and 5C). TBL1X-overexpressing cells were transfected with shFlot2 plasmids, which showed that reduced Flot2 levels markedly inhibited TBL1X overexpression-mediated migration and invasion of NPC cells (Figure 5B and 5D). Further, we established a mouse NPC lung metastasis model by injecting 5-8Fcontrol, 5-8FshTBL1X, and 5-8FshTBL1X+oeFlot2 cells by the tail vein injection. The number of pulmonary metastatic nodules was significantly lower in the 5-8FshTBL1X group than in the 5-8Fcontrol group, and this number was markedly higher in the 5-8FshTBL1X+oeFlot2 group than in the 5-8F shTBL1X group (Figure 5E-F). The HE staining was consistent with this result (Figure 5G).

### Flot2 regulates TBL1X expression through c-myc

**Figure 6:**
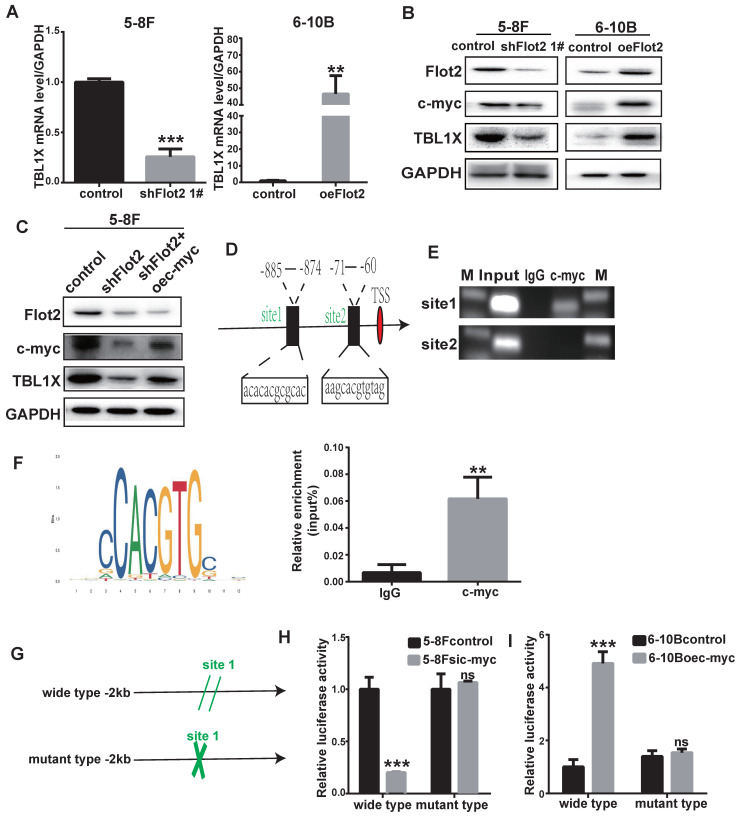
** TBL1X transcription is regulated by Flot2 via c-myc**. (A) qRT-PCR results showed that Flot2 regulated the mRNA level of TBL1X. (B) Western blotting results showed that Flot2 regulated the expression of c-myc and TBL1X. (C) After overexpression of c-myc in 5-8FshFlot2 cells, the TBL1X protein level increased compared to 5-8FshFlot2 cells. (D) The c-myc binding sites in TBL1X promoter region were predicted by using JASPAR. (E, F) ChIP assay identified the binding between c-myc and TBL1X promoter DNA fragment. (G) The wild type and mutant pGL3basic-TBL1X promoter-reporter constructs. (H, I) Relative luciferase activities in 5-8F cells treated with si-c-myc (H) and in 6-10B cells treated with oe-c-myc (I) after transfection of pGL3basic-TBL1X plasmids containing wild type or mutant TBL1X promoter region. The results are shown as means±SD.*P<0.05, **P<0.01, ***P<0.001.

In our previous study, we identified differentially expressed mRNAs in Flot2-depleted NPC cells using a cDNA microarray (GSE67456). The TBL1X gene was one of the downregulated genes in Flot2-knockdown NPC cells. By using qRT-PCR and western blotting methods, we showed that Flot2 knockdown mediated by two different siFlot2 sequences reduced TBL1X expression level, whereas overexpressing Flot2 increased TBL1X level. (Figure 6A-B, Supplementary Figure 3). c-myc is upregulated in NPC^22^. Our results indicated that c-myc expression was reduced after Flot2 knockdown, and Flot2 overexpression increased c-myc protein level (Figure 6B). In 5-8FshFlot2 cells, c-myc overexpression increased TBL1X expression (Figure 6C). c-myc is an important transcription factor. To further investigate whether c-myc directly regulates TBL1X expression, we searched possible binding sites of c-myc in the promoter region of TBL1X gene using the UCSC, PROMO, and JASPAR databases. Two main sites were identified, i.e., site 1 (-885 to -874) and site 2 (-71 to -60) (Figure 6D). We then used a ChIP assay to verify their binding to c-myc, and showed that c-myc was more likely to bind to site 1 region than site 2 region (Figure 6E). ChIP results showed that relative TBL1X enrichment was significantly higher in complex precipitated by anti-c-myc than precipitated by IgG (Figure 6F). We then constructed a TBL1X promoter-luciferase reporter plasmid by inserting the 2 kb of 5' promoter region of TBL1X into the pGL3-basic plasmid as well as a version with mutated c-myc binding sites (Figure 6G). Luciferase activity was significantly reduced in 5-8F cells transfected with wild-type plasmids after c-myc depletion, but not in cells with mutant-type plasmids (Figure 6H). c-myc overexpression increased luciferase activity in 6-10B cells transfected with wild-type plasmids, but not in cells transfected with mutant plasmids (Figure 6I). Taken together, these results demonstrated that Flot2 increased TBL1X expression at the transcriptional level by regulating the expression of c-myc, which is the transcription factor of TBL1X.

**Figure 7:**
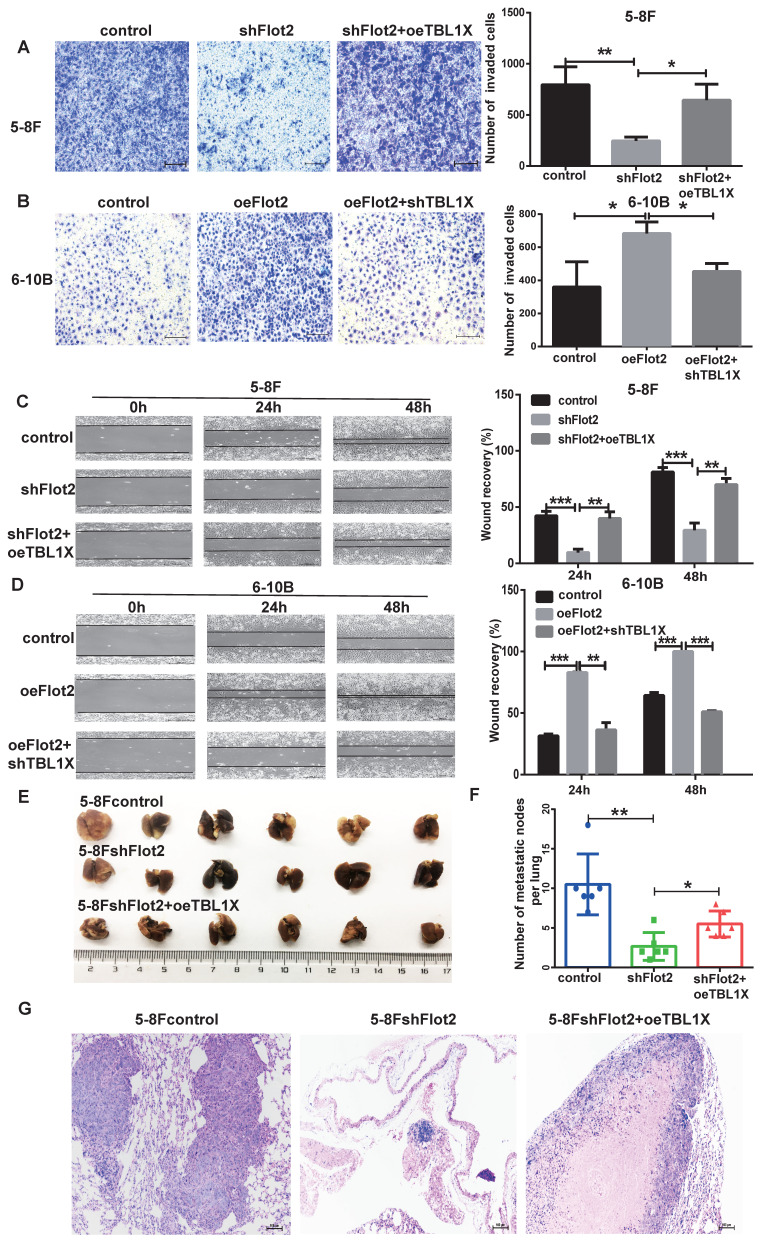
** TBL1X rescues the effect of altered Flot2 expression on NPC cell migration and invasion**. (A) Matrigel invasion analysis showed that oeTBL1X enhanced the invasion of 5-8FshFlot2 cells. (B) shTBL1X inhibited the invasion of 6-10B-Flot2 cells. (C) Wound-healing assay showed that oeTBL1X enhanced the migration 5-8FshFlot2 cells. (D) shTBL1X inhibited the migration of 6-10B-Flot2 cells. (E) Nude mouse metastasis assay showed the lung metastases via tail vein injection of 5-8Fcontrol, 5-8FshFlot2 or 5-8FshFlot2+oeTBL1X cells (n=6, respectively). (F) The number of lung metastases in mice. (G) Representative HE staining images of lung metastases resulting from 5-8Fcontrol, 5-8FshFlot2 or 5-8FshFlot2+oeTBL1X cell inoculations. The results are shown as means±SD. *P<0.05, **P<0.01, ***P<0.001.

Considering TBL1X is regulated by the Flot2-c-myc axis, and c-myc can directly regulate TBL1X expression as a transcription factor, we thus investigated whether TBL1X can rescue NPC cell functions after altering Flot2 levels. 5-8FshFlot2 cells were transfected with TBL1X expression plasmids, the results indicated a significantly higher invasion and migration abilities of 5-8FshFlot2+oeTBL1X cells than those of 5-8FshFlot2 cells (Figure 7A and 7C). Furthermore, 6-10B-Flot2 cells were transfected with shTBL1X plasmids, and reduction of TBL1X expression markedly reduced Flot2 overexpression-mediated migration and invasion abilities of NPC cells (Figure 7B and 7D). Meanwhile, we found that the metastatic lung nodules in nude mice of the shFlot2 group were significantly less than those in controls, which were partly increased in the shFlot2+oeTBL1X group (Figure 7E-F). The HE staining was consistent with this result (Figure 7G). The above results suggested that the TBL1X-Flot2 feedback axis promoted NPC metastasis *in vivo*.

### TBL1X expression is positively correlated with Flot2 expression in NPC

**Figure 8:**
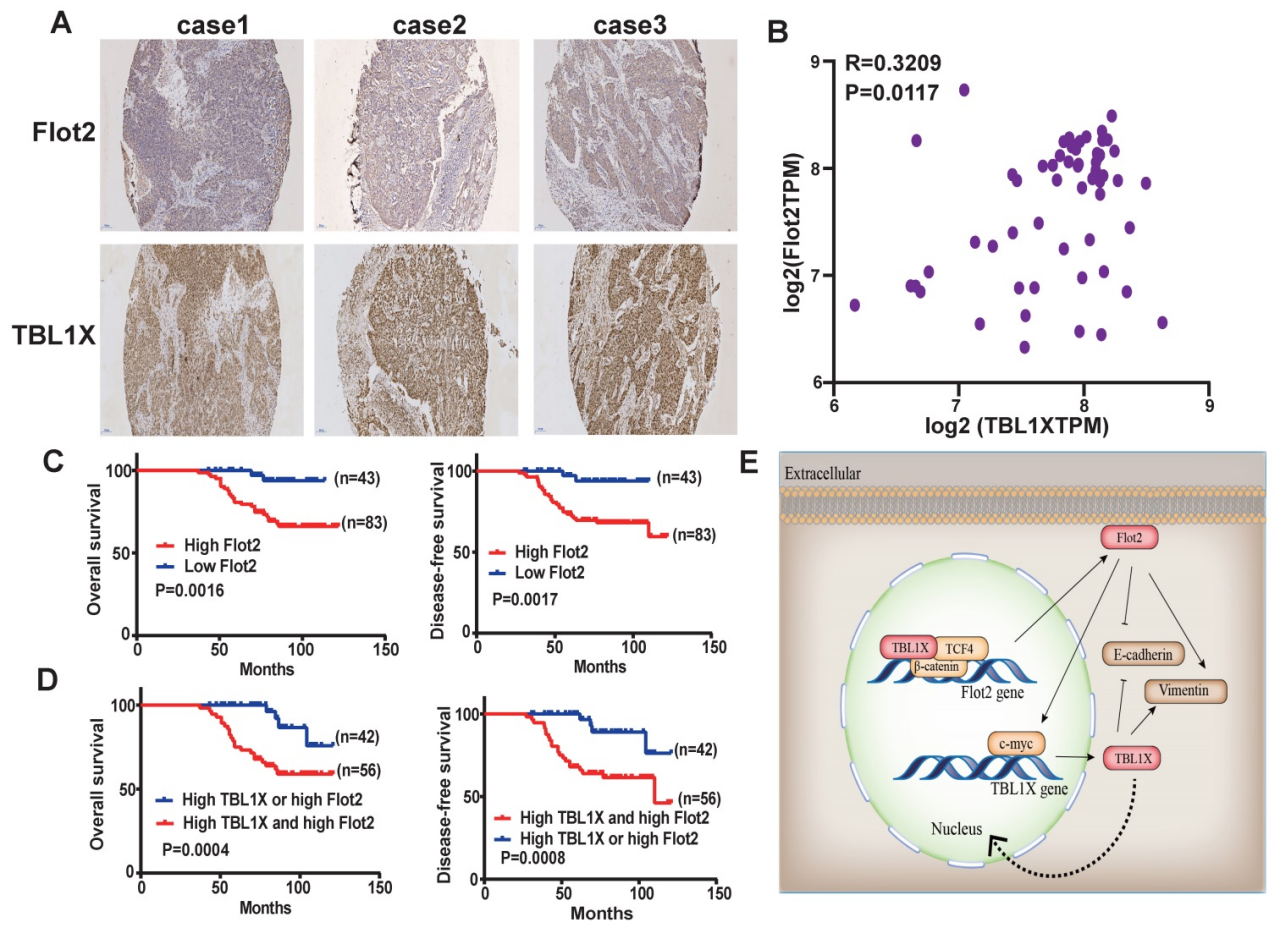
** TBL1X expression is positively associated with Flot2 level in NPC. (**A) Representative IHC images of TBL1X and Flot2 expression in NPC samples (Scale bar=100 μm). (B) Positive correlation between TBL1X and Flot2 expression in NPC tissues of GEO batch. (C) Kaplan Meier survival analysis for NPC patients based on expression levels of Flot2. (D) Kaplan Meier survival analysis for NPC patients based on expression levels of both TBL1X and Flot2. (E) Model for tumor promotion role of TBL1X-Flot2 feedback axis and the underlying mechanisms in NPC.

The above results suggested a TBL1X-Flot2 feedback axis that could influence NPC metastasis. We then investigated the potential prognostic values of the TBL1X-Flot2 feedback axis. Firstly, we examined the correlations of TBL1X and Flot2 in NPC. IHC results showed both high TBL1X and Flot2 levels in NPC tissues (Figure 8A). A Spearman test showed that TBL1X was positively correlated with Flot2 in NPC samples of the GEO batch (61 NPC samples) (R=0.3209;* P*=0.0117) (Figure 8B). Based on the IHC score and Kaplan-Meier survival analyses, NPC patients with high expression of Flot2 showed reduced OS and DFS (Figure 8C). Flot2 expression level was significantly correlated with the tumor node metastasis (TNM) and tumor metastasis; no significant correlation between Flot2 level and gender or age was observed (Table 1). More importantly, NPC patients with both high TBL1X and high Flot2 expression showed more significantly reduced OS and DFS compared to patients with the high level of only one of the two proteins (Figure 8D).

## Discussion

Metastasis is the major cause of treatment failure of NPC patients; thus, investigating the potential molecular mechanisms underlying NPC metastasis and identifying novel effective targets are critical to improving treatment outcomes. In the current study, we identified a novel TBL1X-Flot2 feedback axis that contributed to NPC metastasis. High expression levels of TBL1X and Flot2 predicted unfavorable outcomes in NPC cases. Supported by the experimental data, we demonstrated the clinical significance of TBL1X and Flot2 as biomarkers of NPC metastasis, which supports their high targeted therapeutic value for NPC patients.

Our results showed that TBL1X acted as an oncogene in NPC cases. TBL1X exhibited a higher expression level in metastatic NPC tissues. Upregulated TBL1X expression promoted NPC metastasis. As a transcriptional cofactor, TBL1X acts as a master regulator of Wnt signaling by forming complexes with β-catenin and the TCF family, and guides complexes to the promoter regions of target genes for oncogenesis 23. Our results indicated that TBL1X regulated Flot2 expression by binding to Flot2 endogenous promoter with TCF4. Meanwhile, a previous study showed that TBL1X regulated the stability of critical oncogenic proteins such as PLK1, MYC, and beclin-1 through its interaction with an SKP1-CUL1-F-box (SCF) protein super-complex without β-catenin 24. BC2059, also known as Tegavivint, a disruptor of TBL1X binding, can overcome resistance to BET inhibitors and improve survival in human osteosarcoma and AML 8, 25, which suggests that BC2059 may be an effective drug for relevant tumor treatment. TBL1X is also necessary for RELA recruitment to NF-κB-targeted gene promoters in response to TNF-α 26, which suggests an extra function of TBL1X as a transcriptional activator. In another study, SUMOylation of TBL1 enhances relation with NF-κB in response to TNF-α to promote the expression of targeted genes 27. A similar study has shown that TBL1X suppresses E-cadherin expression by interacting with TWIST, thereby promoting tumor metastasis 7. Therefore, identifying proteins interacting with TBL1X may help elucidate novel functions and mechanisms of TBL1X in tumors. In this study, we first found TBL1X regulated Flot2 expression at both mRNA and protein levels and subsequently discovered TCF4 was one of the potential transcription factors of Flot2. Our results demonstrated that TBL1X regulated Flot2 expression by interacting with TCF4.

Our cDNA microarray (GSE67456) and experimental data proved Flot2 regulated TBL1X expression; thus, the TBL1X-Flot2 axis forms a positive feedback axis and promotes NPC metastasis. Moreover, Flot2 regulated TBL1X expression through c-myc. Liu et al. found that Flot2 regulated c-myc expression by suppressing miR-33b-5p 28. As a membrane protein, Flot2 promotes multiple tumor progression processes through different pathways such as Raf/MEK/ERK1/2^18^, NF-κB, PI3K/AKT 21, and TGF-β 16, 29 pathways. However, the exact mechanisms by which Flot2 activates these oncogenic functions and pathways require further research. Our previous study showed that Flot2 and PLCD3 interact with each other, this complex promotes NPC progression, and PLCD3 is a pivotal enzyme in the phosphoinositide pathway 30. Flotillin-1 (Flot1) is a highly conserved homologous protein of Flot2, and these two proteins form heterotetramers on the cytoplasmic side of the plasma membrane 12. Sumoylation of Flot1 facilitates its nuclear translocation, stabilizes Snail, and promotes the Snail-mediated tumor EMT process 31. This suggests that post-translational modifications could also affect the functions of Flot2 in diseases. In addition to TBL1X, Flot2 is also regulated by several miRNAs, such as miR-34a, miR-133, and miR-138 et al^32^-^34^. In a word, the complex relation between TBL1X and Flot2 may provide the rationale for combination strategies for NPC therapy.

In summary, the TBL1X-Flot2 axis forms a positive feedback loop and promotes metastasis in NPC. High expression levels of both genes are associated with the poor prognosis of NPC cases. Therefore, the TBL1X-Flot2 feedback axis may be a potential target and valuable for improving NPC treatment.

## Supplementary Material

Supplementary figures.

## Figures and Tables

**Table 1:** Association of TBL1X and Flot2 expression with the clinicopathological characteristics of NPC patients

Group	n	TBL1X expression		χ^2^	*P*	n	Flot2 expression		χ^2^	*P*
		low(55)	high(71)				low(43)	high(83)		
**Age**										
<52	58	23	35	0.698	0.404	58	21	37	0.207	0.649
≥52	68	32	36			68	22	46		
**Gender**										
Male	81	37	44	0.379	0.538	81	26	55	0.415	0.519
Female	45	18	27			45	17	28		
**TNM stage**										
Ⅰ+Ⅱ	77	45	32	17.61	*P*<0.001^a)^	77	32	45	4.864	0.027^a)^
Ⅲ+Ⅳ	49	10	39			49	11	38		
**Metastasis**										
Yes	50	9	41	22.174	*P*<0.001^a)^	50	11	39	5.4223	0.02^a)^
No	76	46	30			76	32	44		

a): The values between groups have a statistically significant difference

## Abbreviations

BLCA: bladder urothelial carcinoma
BRCA: breast invasive carcinoma
CHOL: cholangiocarcinoma
COAD: colon adenocarcinoma
ESCA: esophageal carcinoma
GBM: glioblastoma multiforme
HNSC: head and neck squamous cell carcinoma
KICH: kidney chromophobe
KIRC: kidney renal clear cell carcinoma
KIRP: kidney renal papillary cell carcinoma
LGG: brain lower grade glioma
LIHC: liver hepatocellular carcinoma
LUAD: lung adenocarcinoma
LUSC: lung squamous cell carcinoma
PAAD: pancreatic adenocarcinoma
PRAD: prostate adenocarcinoma
READ: rectum adenocarcinoma
STAD: stomach adenocarcinoma
THCA: thyroid carcinoma
UCEC: uterine corpus endometrial carcinoma
TBL1X: Transducin β-like protein 1 X
EMT: epithelial-mesenchymal transition
ZEB1: zinc finger E-box binding homeobox 1
CDH1: cadherin 1
TCF4: transcription factor 4
SSH: suppression subtractive hybridization
IHC: immunohistochemistry
HE: hematoxylin and eosin
qRT-PCR: quantitative real-time reverse transcription PCR
ChIP: chromatin immunoprecipitation assay
Co-IP: Co-immunoprecipitation
OS: overall survival
DFS: disease-free survival
GSEA: gene set enrichment analysis
