# Supplementary material for: TBL1X and Flot2 form a positive feedback loop to promote metastasis in nasopharyngeal carcinoma
Source: Int J Biol Sci. 2022 Jan 1;18(3):1134–49. doi: 10.7150/ijbs.68091 (PMC8771836; doi:10.7150/ijbs.68091)
Supplement: Supplementary file 1 — Supplementary figures. [file ijbsv18p1134s1.pdf]

Supplementary Figure 1: Depletion of TBL1X with shTBL1X 2# reduces NPC cell migration and invasion. (A) Transwell invasion assay. (B) Wound-healing assay. (C) Western blotting results indicated the effects of TBL1X interference on EMT markers. The results are shown as means±SD. *$P<0.05$, **$P<0.01$, ***$P<0.001$.

Supplementary Figure 2. The effects of depletion of TBL1X and TCF4 with the second siRNA on Flot2 level. (A) Western blotting results indicated the effect of TBL1X interference on Flot2 level. (B) Western blotting results indicated the effect of TCF4 interference on Flot2 level.

Supplementary Figure 3. The effects of depletion of Flot2 with the second siRNA on c-myc and TBL1X level. Western blotting results indicated the effect of Flot2 interference on c-myc and TBL1X level.

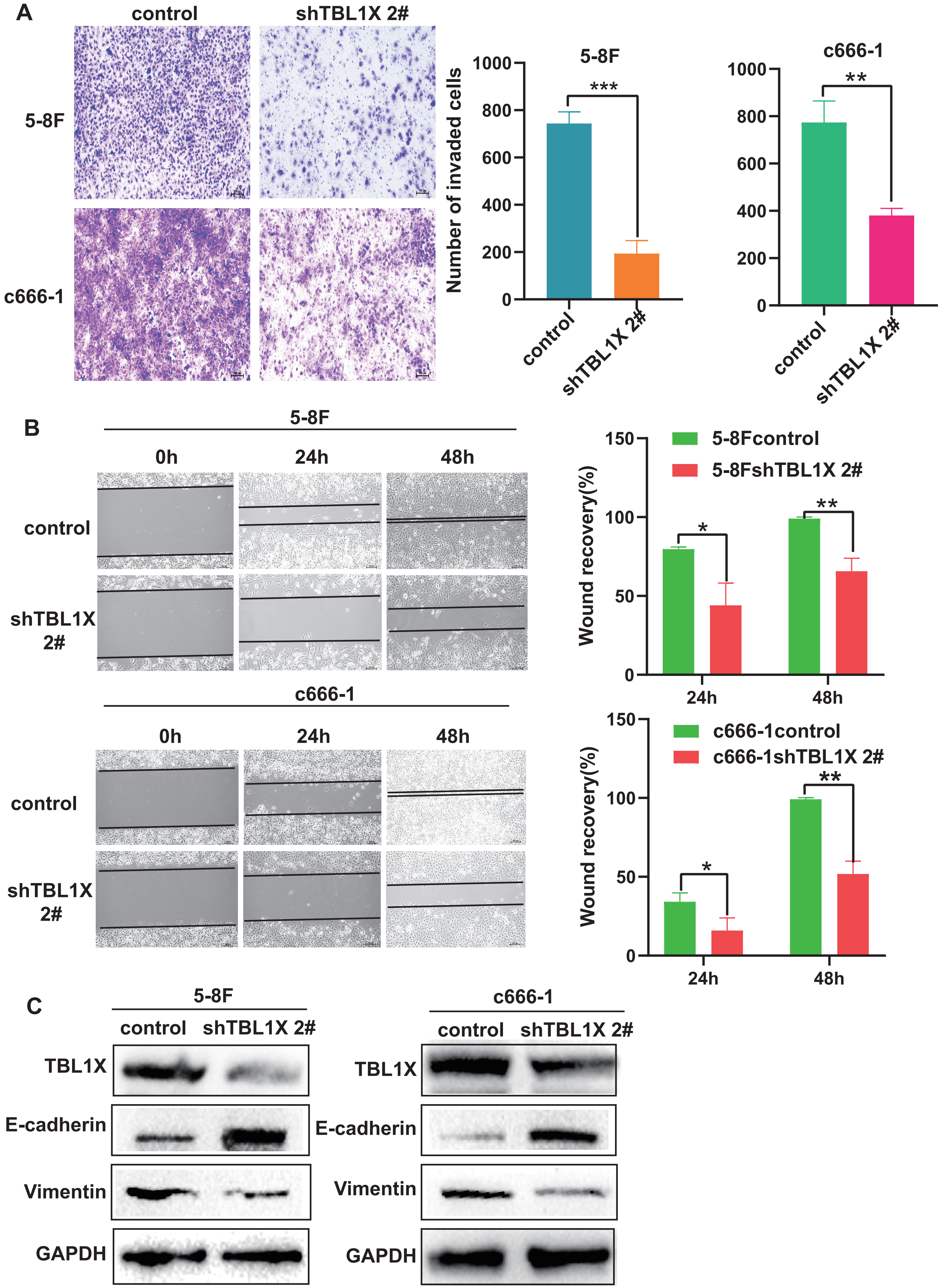
A
control
shTBL1X 2#
5-8F
c666-1
Number of invaded cells
5-8F
***
c666-1
**
1000
800
600
400
200
0
control
shTBL1X 2#
B
5-8F
0h
24h
48h
control
shTBL1X 2#
c666-1
5-8Fcontrol
5-8FshTBL1X 2#
Wound recovery(%)
150
100
50
0
*
**
24h
48h
c666-1control
c666-1shTBL1X 2#
C
5-8F
c666-1
TBL1X
E-cadherin
Vimentin
GAPDH

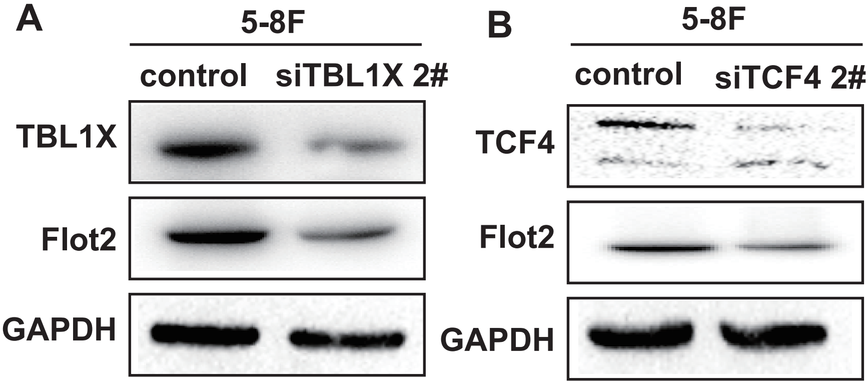
A
5-8F
control
siTBL1X 2#
TBL1X
Flot2
GAPDH
B
5-8F
control
siTCF4 2#
TCF4
Flot2
GAPDH

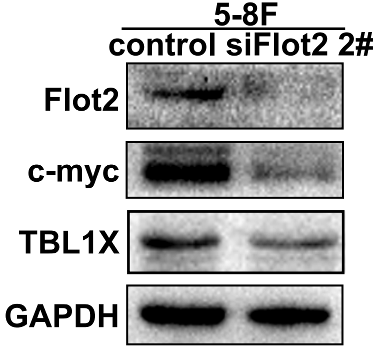
5-8F
control
siFlot2 2#
Flot2
c-myc
TBL1X
GAPDH
